# Gender Differences in the Outcome of Offspring Prenatally Exposed to Drugs of Abuse

**DOI:** 10.3389/fnbeh.2020.00072

**Published:** 2020-06-05

**Authors:** Francesco Traccis, Roberto Frau, Miriam Melis

**Affiliations:** Department of Biomedical Sciences, Division of Neuroscience and Clinical Pharmacology, University of Cagliari, Cagliari, Italy

**Keywords:** development, drugs of abuse, gender, neuropsychiatric, prenatal, sex, vulnerability

## Abstract

Despite great efforts to warn pregnant women that drugs of abuse impact development of the embryo and the fetus, the use of legal and illegal drugs by childbearing women is still a major public health concern. In parallel with well-established teratogenic effects elicited by some drugs of abuse, epidemiological studies show that certain psychoactive substances do not induce birth defects but lead to subtle neurobehavioral alterations in the offspring that manifest as early as during infancy. Although gender differences in offspring susceptibility have not been fully investigated, a number of longitudinal studies indicate that male and female progeny exposed *in utero* to drugs of abuse show different vulnerabilities to deleterious effects of these substances in cognitive, executive, and behavioral domains. Here, we briefly review the existing literature focusing on gender differences in the neurobehavioral consequences of maternal exposure to drugs of abuse. Overall, the data strongly indicate that male exposed progeny are more susceptible than female to dysfunctions in cognitive processing and emotional regulation. However, insights into the mechanisms determining this natural phenomenon are not currently available. Our analysis prompts future investigations to implement clinical studies including the influence of gender/sex as a biological variable in the outcome of offspring prenatally exposed to drugs of abuse.

## Introduction

As a rule, drugs should not be used during pregnancy unless prescribed, because many can be toxic to the placenta or the developing fetus. Yet, the use of drugs, including prescription or non-prescription drugs, medicinal herbs, and licit (tobacco and alcohol) or illicit drugs, during pregnancy keeps increasing (SAMHSA, 2011). Indeed, objective measurements of xenobiotics in meconium, amniotic fluid, and cord blood indicate widespread fetal exposure to such agents during their intrauterine life (for an excellent review see Barr et al., 2007). Such exposure may induce developmental adaptations that can be interpreted as derangements from normal development, which not only interfere with the immediate viability of the fetus but may also result in the individual’s adverse health outcome in the short and long term (Hales and Barker, 2001; Barker, 2007). Hence, the “developmental origin of health and disease” hypothesis (Barker, 2007) stems from epidemiological studies showing that malnutrition, exposure to xenobiotics (e.g., environmental chemicals and prescription, legal, and illegal drugs), infective diseases, or stress during specific periods of development might increase the risk of disorders later in life. This hypothesis also stresses the importance of investigating the mechanisms of fetal exposure to xenobiotics and further in general to adverse intrauterine and perinatal factors.

In this minireview, we will provide an up-to-date analysis of the evidence for a sex differential in the susceptibility to the consequences of maternal drug use on neurocognitive and behavioral development of the offspring. Research has pointed to gender differences in these sequelae, since exposed males often appear more vulnerable than exposed females. Insights into the neurobiological mechanisms underlying the sex bias observed in certain neurobehavioral outcomes remain unidentified. At this stage, we could only make inferences from animal studies, although they do not allow for a precise understanding of the underpinnings, especially in the context of sex differences. In particular, many factors might moderate the reported sex dichotomy, including individual (e.g., species, strain, age) and experimental (e.g., design, drug, dosage, route, regimen) variables, and objective endpoints (e.g., behavioral paradigm, experimental technique). Here, we attempt to integrate the gender difference results across drugs used by pregnant women. Such integration could be useful for physicians and healthcare providers when caring for a pregnant substance abusing woman. Interspecies extrapolations will be carefully avoided to ensure sound conclusions. The authors refer to excellent preclinical studies’ reviews (Bruin et al., 2010; Schneider et al., 2011; Ross et al., 2015; Gkioka et al., 2016; Comasco et al., 2018; Scheyer et al., 2019).

## Substance Use in Women

The historical gap in substance use prevalence between men and women has gradually narrowed in the past decade, particularly among adolescents (Keyes et al., 2008; Seedat et al., 2009; Steingrimsson et al., 2012; EMCDDA, 2019). While women still exhibit lower rates of drug use disorder than men, prevalence rates indicate that the number of female drug abusers is on the rise. A recent snapshot of the European drug use situation shows that women account for one-quarter of the general population with drug issues and around one-fifth of all first-time drug abuse treatment seekers (EMCDDA, 2019). Gender differences are clear in the pattern of use at each stage of the addiction cycle. Women typically begin to use substances later in life (Greenfield et al., 2010; Keyes et al., 2010), misuse prescription drugs (e.g., opioids) (McHugh et al., 2013), and their rate of consumption increases more rapidly than that of men (Greenfield et al., 2010; Keyes et al., 2010). Women also exhibit higher prevalence rates of comorbidity with other psychiatric disorders as well as of relapse (Wilcox and Yates, 1993; Conway et al., 2006; Back et al., 2011; Khan et al., 2013).

## Drug Use During Pregnancy and Breastfeeding: Effects on Male and Female Offspring

The consumption of drugs in childbearing women has been progressively increasing. Women abusing recreational drugs before pregnancy tend to continue the use even during gestation (Forray, 2016), and this use is not limited to illegal drugs but includes prescription and over-the-counter drugs. Approximately 60% of pregnant women take prescription drugs and about 13% of them use herbal supplements. Furthermore, the infographics based on the National Survey on Drug Use and Health (SAMHSA, 2018) show that 5.4% of pregnant women have used illicit drugs in the past 30 days, while 9.9 and 11.6% reported past-month alcohol or cigarette smoking use, respectively. To complicate this issue, many women take drugs when they are not aware of being pregnant.

Regardless of their legal status, all drugs cross and/or alter the placental barrier, reach the fetus, and affect infant development. Additionally, multiple drugs also pass into mother’s breast milk, thus resulting in prolonged drug exposure of the newborn. According to the United States Centers for Disease Control and Prevention, almost 3% of newborns have birth defects because of genetic, environmental, or other unknown causes (Parker et al., 2010). Among environmental factors, drug use is the major cause leading to birth defects ranging from fetal growth reductions to medical complications such as preterm birth and infections. Furthermore, the progeny prenatally exposed to drugs of abuse develop neurobehavioral phenotypes that manifest during infancy and persist to adolescence and young adulthood. Research on the effects of prenatal alcohol, tobacco, opioids, stimulants, and cannabis indicates an association between fetal exposure to these substances and deficits in cognitive and behavioral domains. However, in humans, the role of fetal sex on functional consequences of prenatal exposure to drugs of abuse remains grossly understudied. Here we present data on illicit psychostimulants, opioids, cannabis, nicotine, and alcohol in an attempt to provide a clear picture of neurobehavioral outcomes in male and female progeny. When gender differences have not been examined, our interpretation is limited to the overall outcome.

### Effects of *in utero* Exposure to Psychostimulants

**FIGURE 1:**
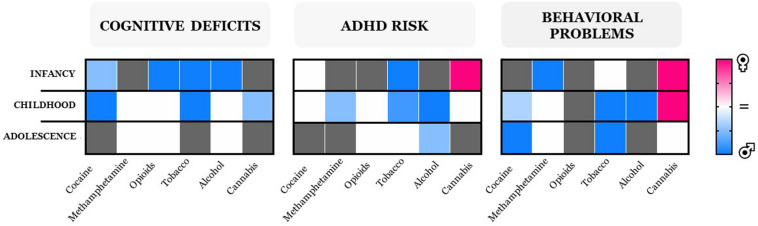
Heatmaps of neurobehavioral outcomes collectively identified as cognitive deficits (including executive function), ADHD risk, and behavioral problems, with missing information on gender differences shown in gray. Outcome intensities are displayed as colors ranging from pink (female) to blue (male) shown in the key. Gender differences were not observed in executive functions during childhood in offspring prenatally exposed to cocaine in one study only (Accornero et al., 2007); therefore, the heatmap displays blue as a result of the larger number of consistent studies reporting that boys are more susceptible to exhibit deficits in cognitive abilities when compared to girls (see text and also Table 1).

Psychostimulants, including cocaine and methamphetamine, are the illicit drugs most commonly used by childbearing women, though no recent estimate of their consumption during pregnancy is known. Despite their well-described neurotoxic effects on central nervous system (CNS) development, only very few studies have addressed the negative neurobehavioral sequalae on human offspring, particularly when gender is included as an additional biological variable (Table 1 and Figure 1).

**TABLE 1:** Detailed information on the studies covered in this minireview examining gender as a variable.

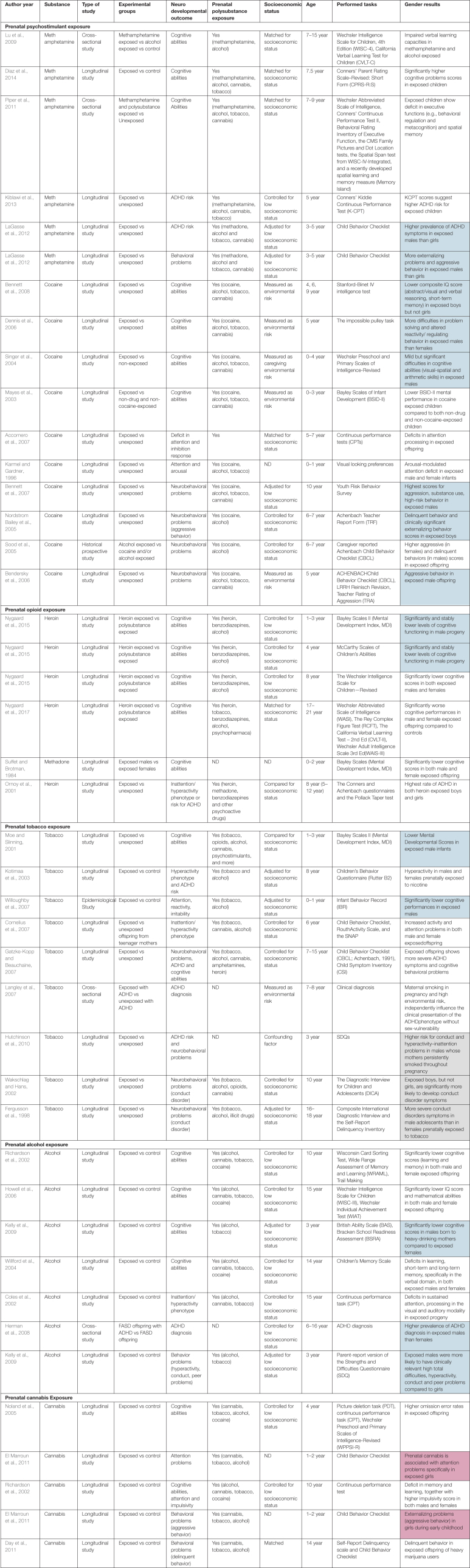

*A thorough description of each study is presented with designed study type, experimental groups enrolled, outcomes, occurrence of poly-drug use, socioeconomic status, age at assessment, type of task used, and gender results. Gender effect is displayed as pink and blue for female and male progeny, respectively. Gender differences not observed are white.*

Longitudinal studies of long-term consequences of cocaine use during pregnancy on the offspring focusing on emotional regulation, behavior, and cognition suggest that female gender is a protective factor (Singer et al., 2004; Dennis et al., 2006; Accornero et al., 2007; Bennett et al., 2008; Ackerman et al., 2010; Bridgett and Mayes, 2011). Male progeny exhibit stronger impairment in inhibitory response, whereas females exhibit only mild alterations that disappear with age (Carmody et al., 2011). Accordingly, male offspring exhibit greater emotion regulation problems and externalizing symptoms (e.g., aggressive and risky behaviors); lower intellectual capabilities; and deficits in attention, short-term memory, and problem solving compared to female offspring (Bennett et al., 2002, 2007, 2008; Delaney-Black et al., 2004; Nordstrom Bailey et al., 2005; Sood et al., 2005; Bendersky et al., 2006; Dennis et al., 2006; Carmody et al., 2011). In contrast, no gender dichotomy was found in the occurrence of attention deficit/hyperactivity disorder (ADHD) phenotypes from infancy to preadolescence (Karmel and Gardner, 1996; Mayes, 1996; Mayes et al., 1998, 2003; Accornero et al., 2007).

With regard to methamphetamine, the most frequent outcomes reported in newborns occur during the first year of life and include motor dysfunctions (e.g., disorganized behaviors with poor quality of movement), which tend to disappear with development in boys (LaGasse et al., 2012; Shah et al., 2012; Zabaneh et al., 2012; Kiblawi et al., 2014), whereas they persist throughout adolescence in girls (Eriksson and Zetterström, 1994; Cernerud et al., 1996). In contrast, other neurobehavioral problems (e.g., anxious/depressive phenotypes, emotional problems) appear during late infancy and childhood and do not exhibit sex bias (LaGasse et al., 2012). Similarly, impairments in cognitive skills occur equally in both female and male offspring (Lu et al., 2009; Piper et al., 2011; Diaz et al., 2014). However, deficits in inhibitory control and ADHD-like symptoms are prevalent in boys (LaGasse et al., 2012; Kiblawi et al., 2013).

### Effects of Prenatal Exposure to Opioids

Regardless of the efforts aimed at discouraging opioid use, prevalence rates show an increasing trend in pregnant women (Haight et al., 2018). However, gender was not considered in most of the human studies on the effects of heroin, methadone, and other prescription opioids.

Children born to mothers who use opioids during gestation suffer from the so-called neonatal opioid withdrawal syndrome (NOWS) (Gomez-Pomar and Finnegan, 2018), characterized by several signs and symptoms (e.g., tremors, sleep problems, hyperactive reflexes, vomiting, dehydration, and respiratory problems), which are more severe in boys than in girls (Jansson et al., 2007, 2010). Maternal consumption of methadone—the gold standard for opioid maintenance therapy—is associated with poorer cognitive performance and lower IQ scores in exposed males when compared to females during infancy, an age-dependent effect (Suffet and Brotman, 1984; Nygaard et al., 2015). However, no gender difference is found in symptoms related to ADHD and aggressive behavior up to preadolescence (Ornoy et al., 2001).

### Effects of Maternal Tobacco

Nicotine and its related tobacco products are the most studied substances in relation to long-term neurobehavioral outcomes in offspring exposed to tobacco during pregnancy. Despite the limitations due to several environmental confounding factors, a high degree of consistency exists for the association of maternal smoking and cognitive and behavioral problems (for an exhaustive review, see England et al., 2017). From these studies emerge a male bias toward diverse behavioral and cognitive domains, depending on age: at 6–8 months, males appear more vulnerable to deficits in cognitive and executive functions (e.g., inattention) and in motor functions and to alterations in reactivity (Moe and Slinning, 2001; Wakschlag and Hans, 2002; Willoughby et al., 2007). From infancy through childhood, boys appear at risk for ADHD (Kotimaa et al., 2003; Cornelius et al., 2007; Willoughby et al., 2007; Agrawal et al., 2010; Hutchinson et al., 2010); however, only during infancy do they display less positive mood (Pickett et al., 2008) than females; during childhood and adolescence, males present more externalizing and disruptive behaviors (e.g., conduct disorders, antisocial behavior) than females (Wakschlag et al., 1997; Fergusson et al., 1998; Hutchinson et al., 2010). Conversely, parental tobacco exposure is associated with nicotine dependence and high consumption of tobacco only in adolescent girls (Rydell et al., 2012). Although the risk of developing ADHD symptoms in nicotine-exposed progeny is high during adolescence, no gender differences were found (Gatzke-Kopp and Beauchaine, 2007; Agrawal et al., 2010; Sourander et al., 2019).

### Effects of Maternal Alcohol

Despite the widely described dose-dependent teratogenic effect of alcohol (Kodituwakku, 2007; Ornoy and Ergaz, 2010), approximately 10% of women aged between 15 and 44 years consume alcohol during pregnancy, with 3% exhibiting a binge-drinking pattern (SAMHSA, 2011). Irrespective of the amount and pattern of consumption, a wealth of clinical evidence describes that prenatal alcohol exposure markedly impairs cognitive, behavioral, and motor functions of offspring (Mattson et al., 1998; Coles et al., 2002; Richardson et al., 2002; Willford et al., 2004; Riley and McGee, 2005; Howell et al., 2006). Maternal moderate to heavy drinking produces a group of pathological conditions termed fetal alcohol spectrum disorder (FASD). Epidemiological studies report sexual dichotomy in FASD, with prevalence rates and severity being higher in male than in female patients (May et al., 2007; Astley, 2010; Thanh et al., 2014; but see May et al., 2014; Fox et al., 2015). A sex bias is also described for other psychopathological traits, such as elevated rates of ADHD in 6- to 16-year-old boys but not girls (Coles et al., 2002; Herman et al., 2008). Boys also exhibit altered responses to stress, measured as larger changes in cortisol levels induced by stress-related cues (Haley et al., 2006). In contrast, neuroimaging studies do not reveal sex differences in long-term abnormalities of brain morphology because the reduction in both size and volume of frontal, temporal, cingulate, and striatal regions of offspring prenatally exposed to alcohol did not differ between genders (Eckstrand et al., 2012; Treit et al., 2013; De Guio et al., 2014). These findings suggest that such psychopathological traits cannot be attributed to these structural changes.

### Effects of *in utero* Cannabis Exposure

In line with the data on general population, the rates of cannabis use among pregnant women have markedly increased, with prevalence rates reaching 75% between 2002 and 2016 (Brown et al., 2017). Despite this alarming scenario, a few studies have assessed the long-term neurobehavioral repercussions of maternal cannabis use on the offspring, though gender differences were not consistently examined: the *Ottawa Prenatal Prospective Study* (OPPS), the *Maternal Health Practices and Child Development Project* (MHPCDP), the *Generation R* study, and *Adolescent Brain Cognitive Development* (ABCD) study. The OPPS study included gender as a confounding factor, and it described a number of long-lasting neurobehavioral alterations, ranging from heightened tremors and startle responsiveness to deficits in executive function (e.g., attention, cognitive flexibility, problem solving, impulse control) (Fried and Makin, 1987; Fried and Smith, 2001). Similarly, gender was not examined when assessing performance in memory, verbal, and perceptual processes as well as the first clinical signs of impulsivity at childhood (Smith et al., 2006). However, when the same authors subsequently included the gender factor on clinical signs that persisted at young adulthood, such as deficits in executive function tasks that require impulse control, they found no gender differences (Smith et al., 2004). In the MHPCD study, the authors seldom included “gender” in their analysis. However, they reported (1) significant sleep disturbances and deficits in mental development as well as in short-term memory and verbal reasoning at both 9 months and 3 years of age; (2) deficits in attention and memory, increased anxiety/depressive symptoms, impulsivity, hyperactivity, and aggression at 6 and 14 years of age (Richardson et al., 1989, 2002; Dahl et al., 1995; Leech et al., 2006; Day et al., 2011). Gender at 10 years of age did not affect cognitive deficits (Richardson et al., 2002). In contrast, the Generation R study showed that girls but not boys at 18 months of age exhibited increased scores on an aggressive behavior scale that persisted through childhood (El Marroun et al., 2011). Notably, this sex bias disappears during adolescence. Also, during infancy girls appear to be at risk for the development of ADHD, a susceptibility that is age dependent (Table 1 and Figure 1). Remarkably, although from Generation R and ABCD studies maternal cannabis use has been associated to proneness to psychosis in middle to late childhood, significantly earlier than the typical onset of first psychotic episode (Bolhuis et al., 2018; Fine et al., 2019; Paul et al., 2019), again gender was not considered. Importantly, an independent investigation showed that prenatal marijuana exposure has an equally negative effect on sustained attention of the offspring from childhood to adolescence (Noland et al., 2005).

## Conclusion

The literature here examined reveals gender differences in immediate and long-term negative consequences of maternal drug use on both cognition and behavior. When gender was included as a variable, irrespective of the drug used, male progeny appear more vulnerable to cognitive deficits and at risk of ADHD from infancy through childhood (Table 1 and Figure 1). Notably, these gender differences tend to disappear with age. However, we cannot depict a clear picture for internalizing problems, drug use, and motor function deficits due to the paucity of data. Regarding the problems in the behavioral domain (i.e., externalizing problems), the current scenario is clearer: girls exposed *in utero* to cannabis are more vulnerable than boys up until adolescence, but this conclusion cannot be extended to other drugs. Remarkably, this is in contrast to what is often reported in rodent studies (Fernandez-Ruiz et al., 1998; Hurd et al., 2019; Scheyer et al., 2019; de Salas-Quiroga et al., 2020), where female sex often acts as a protective factor. Nevertheless, the advantage of animal studies is to dissect the effects of genetic, biological, and/or environmental risk factors. The establishment of a biological causality between prenatal drug exposure and repercussions on the progeny from animal investigations is pivotal. These mechanistic insights along with the observations reported in human studies may help in developing therapeutic interventions, on a gender-specific basis, which would ultimately result in more effective treatment outcome.

The longitudinal studies examined have often considered different factors that might have contributed to gender differences, including socioeconomic status, lifestyle indicators, stressful life events, social support (or lack thereof), and psychiatric comorbidity. In this regard, an additional degree of complexity arises from the evidence that single drug use is virtually non-existent. At this stage, we cannot certainly resolve this issue in human studies, as it deserves as much attention as neuroimaging and omics analyses to reveal neurobiological underpinnings of drug-exposed phenotypes. Of similar importance is the need to study the association between the perturbations of *in utero*–placental exchange and adverse mental health outcome later in life. Indeed, increasing evidence points to the role of the placenta in fetal programming, which is altered in response to prenatal insults and contributes to psychopathology (Burton et al., 2010; Khalife et al., 2012; Roescher et al., 2014; Park et al., 2018; Kratimenos and Penn, 2019). Notably, the placenta influences in a sex-dependent manner the outcome for offspring who were exposed to perinatal malnutrition and stressors (Walsh et al., 2019). However, research into whether the gender bias results from sex differences in placental structure and functions or its genes, proteins, and steroids is surprisingly lacking. Hence, future research should aim at disentangling how sex impacts neurobiology from the transfer of maternal drug concentrations across the placenta to the effect on placental gene transcription or expression of discrete transporters (e.g., ATP-binding cassette carriers) in the cord. In fact, to date, such investigations have been performed only to relate maternal drug use and placental perturbations to fetal growth and other morphological abnormalities (Janssen et al., 2015).

Substance (ab)use screening protocols, including questionnaires and urine toxicology testing, should be established worldwide as routine to identify pregnant women using drugs. Public health interventions regarding the awareness of the harm associated with maternal drug use, and special programs to enter treatment and/or increase spontaneous quit rates, should be implemented (Jantzen et al., 1998; Forray, 2016 and references therein; Patrick et al., 2017). Progress on tailored, safe, and acceptable pharmacotherapies to restore proper neurodevelopmental trajectories of the progeny should be incentivized. Additional preventative outreach programs should be implemented to raise community awareness and support and to provide access to treatment for the children who are prenatally exposed to drugs. Finally, future investigations should be implemented to include the influence of sex as a biological variable (for guidelines please refer to (Clayton, 2018; Mannon et al., 2020) in the outcome of offspring prenatally exposed to drugs of abuse.

## Author Contributions

All authors participated in the conceptualization, design, and preparation of the manuscript.

## Conflict of Interest

The authors declare that the research was conducted in the absence of any commercial or financial relationships that could be construed as a potential conflict of interest.

## References

[B1] Accornero V. H. Amado A. J. Morrow C. E. Xue L. Anthony J. C. Bandstra E. S. (2007). Impact of prenatal cocaine exposure on attention and response inhibition as assessed by continuous performance tests. *J. Dev. Behav. Pediatr.* 28 195–205. 10.1097/01.DBP.0000268560.72580.f9 17565286 PMC2760335

[B2] Ackerman J. P. Riggins T. Black M. M. (2010). A review of the effects of prenatal cocaine exposure among school-aged children. *Pediatrics* 125 554–565. 10.1542/peds.2009-0637 20142293 PMC3150504

[B3] Agrawal A. Scherrer J. F. Grant J. D. Sartor C. E. Pergadia M. L. Duncan A. E. (2010). The effects of maternal smoking during pregnancy on offspring outcomes. *Prev. Med.* 50 13–18. 10.1016/j.ypmed.2009.12.009 20026103 PMC2813884

[B4] Astley S. J. (2010). Profile of the first 1,400 patients receiving diagnostic evaluations for fetal alcohol spectrum disorder at the Washington State Fetal Alcohol Syndrome Diagnostic & Prevention Network. *Can. J. Clin. Pharmacol.* 17 132–164. 20335648

[B5] Back S. E. Payne R. L. Wahlquist A. H. Carter R. E. Stroud Z. Haynes L. (2011). Comparative profiles of men and women with opioid dependence: results from a national multisite effectiveness trial. *Am. J. Drug Alcohol Abuse* 37 313–323. 10.3109/00952990.2011.596982 21854273 PMC3164783

[B6] Barker D. J. (2007). The origins of the developmental origins theory. *J. Intern. Med.* 261 412–417. 10.1111/j.1365-2796.2007.01809.x 17444880

[B7] Barr D. B. Bishop A. Needham L. L. (2007). Concentrations of xenobiotic chemicals in the maternal-fetal unit. *Reprod. Toxicol.* 23 260–266. 10.1016/j.reprotox.2007.03.003 17386996

[B8] Bendersky M. Bennett D. Lewis M. (2006). Aggression at age 5 as a function of prenatal exposure to cocaine, gender, and environmental risk. *J. Pediatr. Psychol.* 31 71–84. 10.1093/jpepsy/jsj025 15827351 PMC1522058

[B9] Bennett D. Bendersky M. Lewis M. (2007). Preadolescent health risk behavior as a function of prenatal cocaine exposure and gender. *J. Dev. Behav. Pediatr.* 28 467–472. 10.1097/DBP.0b013e31811320d8 18091092

[B10] Bennett D. S. Bendersky M. And Lewis M. (2008). Children’s cognitive ability from 4 to 9 years old as a function of prenatal cocaine exposure, environmental risk, and maternal verbal intelligence. *Dev. Psychol.* 44 919–928. 10.1037/0012-1649.44.4.919 18605824 PMC2556289

[B11] Bennett D. S. Bendersky M. Lewis M. (2002). Children’s intellectual and emotional-behavioral adjustment at 4 years as a function of cocaine exposure, maternal characteristics, and environmental risk. *Dev. Psychol.* 38:648. 10.1037/0012-1649.38.5.648 12220044 PMC1522054

[B12] Bolhuis K. Kushner S. A. Yalniz S. Hillegers M. H. Jaddoe V. W. Tiemeier H. (2018). Maternal and paternal cannabis use during pregnancy and the risk of psychotic-like experiences in the offspring. *Schizophr. Res.* 202 322–327. 10.1016/j.schres.2018.06.067 29983267

[B13] Bridgett D. J. Mayes L. C. (2011). Development of inhibitory control among prenatally cocaine exposed and non-cocaine exposed youths from late childhood to early adolescence: the effects of gender and risk and subsequent aggressive behavior. *Neurotoxicol. Teratol.* 33 47–60. 10.1016/j.ntt.2010.08.002 21256424 PMC3052921

[B14] Brown Q. L. Sarvet A. L. Shmulewitz D. Martins S. S. Wall M. M. Hasin D. S. (2017). Trends in marijuana use among pregnant and nonpregnant reproductive-aged women, 2002-2014. *JAMA* 317 207–209. 10.1001/jama.2016.17383 27992619 PMC5595220

[B15] Bruin J. E. Gerstein H. C. Holloway A. C. (2010). Long-term consequences of fetal and neonatal nicotine exposure: a critical review. *Toxicol. Sci.* 116 364–374. 10.1093/toxsci/kfq103 20363831 PMC2905398

[B16] Burton G. J. Jauniaux E. Charnock-Jones D. S. (2010). The influence of the intrauterine environment on human placental development. *Int. J. Dev. Biol.* 54 303–311. 19757391 10.1387/ijdb.082764gb

[B17] Carmody D. P. Bennett D. S. Lewis M. (2011). The effects of prenatal cocaine exposure and gender on inhibitory control and attention. *Neurotoxicol. Teratol.* 33 61–68. 10.1016/j.ntt.2010.07.004 21256425 PMC3052746

[B18] Cernerud L. A. R. S. Eriksson M. Jonsson B. Steneroth G. Zetterstrom R. (1996). Amphetamine addiction during pregnancy: 14-year follow-up of growth and school performance. *Acta Paediatr.* 85 204–208. 10.1111/j.1651-2227.1996.tb13993.x 8640051

[B19] Clayton J. A. (2018). Applying the new SABV (sex as a biological variable) policy to research and clinical care. *Physiol. Behav.* 187 2–5. 10.1016/j.physbeh.2017.08.012 28823546

[B20] Coles C. D. Platzman K. A. Lynch M. E. Freides D. (2002). Auditory and visual sustained attention in adolescents prenatally exposed to alcohol. *Alcohol. Clin. Exp. Res.* 26 263–271. 10.1111/j.1530-0277.2002.tb02533.x 11964567

[B21] Comasco E. Rangmar J. Eriksson U. J. Oreland L. (2018). Neurological and neuropsychological effects of low and moderate prenatal alcohol exposure. *Acta Physiol.* 222:e12892. 10.1111/apha.12892 28470828

[B22] Conway K. P. Compton W. Stinson F. S. Grant B. F. (2006). Lifetime comorbidity of DSM-IV mood and anxiety disorders and specific drug use disorders: results from the national epidemiologic survey on alcohol and related conditions. *J. Clin. Psychiatry* 67 247–257. 10.4088/jcp.v67n0211 16566620

[B23] Cornelius M. D. Goldschmidt L. DeGenna N. Day N. L. (2007). Smoking during teenage pregnancies: effects on behavioral problems in offspring. *Nicotine Tob. Res.* 9 739–750. 10.1080/14622200701416971 17577803 PMC2593871

[B24] Dahl R. E. Scher M. S. Williamson D. E. Robles N. Day N. (1995). A longitudinal study of prenatal marijuana use: Effects on sleep and arousal at age 3 years. *Arch. Pediatr. Adolesc. Med.* 149 145–150. 7849875 10.1001/archpedi.1995.02170140027004

[B25] Day N. L. Leech S. L. Goldschmidt L. (2011). The effects of prenatal marijuana exposure on delinquent behaviors are mediated by measures of neurocognitive functioning. *Neurotoxicol. Teratol.* 33 129–136. 10.1016/j.ntt.2010.07.006 21256427 PMC3052937

[B26] De Guio F. Mangin J. F. Rivière D. Perrot M. Molteno C. D. Jacobson S. W. (2014). A study of cortical morphology in children with fetal alcohol spectrum disorders. *Hum. Brain Mapp.* 35 2285–2296. 10.1002/hbm.22327 23946151 PMC6869611

[B27] de Salas-Quiroga A. García-Rincón D. Gómez-Domínguez D. Valero M. Simón-Sánchez S. Paraíso-Luna J. (2020). Long-term hippocampal interneuronopathy drives sex-dimorphic spatial memory impairment induced by prenatal THC exposure. *Neuropsychopharmacology* 45 877–886. 10.1038/s41386-020-0621-3 31982904 PMC7075920

[B28] Delaney-Black V. Covington C. Nordstrom B. Ager J. Janisse J. Hannigan J. H. (2004). Prenatal cocaine: quantity of exposure and gender moderation. *J. Dev. Behav. Pediatr.* 25 254–263. 10.1097/00004703-200408000-00005 15308926

[B29] Dennis T. Bendersky M. Ramsay D. Lewis M. (2006). Reactivity and regulation in children prenatally exposed to cocaine. *Dev. Psychol.* 42 688–697. 10.1037/0012-1649.42.4.688 16802901 PMC1861810

[B30] Diaz S. D. Smith L. M. LaGasse L. L. Derauf C. Newman E. Shah R. (2014). Effects of prenatal methamphetamine exposure on behavioral and cognitive findings at 7.5 years of age. *J. Pediatr.* 164 1333–1338. 10.1016/j.jpeds.2014.01.053 24630350 PMC4035384

[B31] Eckstrand K. L. Ding Z. Dodge N. C. Cowan R. L. Jacobson J. L. Jacobson S. W. (2012). Persistent dose-dependent changes in brain structure in young adults with low-to-moderate alcohol exposure in utero. *Alcohol. Clin. Exp. Res.* 36 1892–1902. 10.1111/j.1530-0277.2012.01819.x 22594302 PMC3424348

[B32] El Marroun H. Hudziak J. J. Tiemeier H. Creemers H. Steegers E. A. Jaddoe V. W. (2011). Intrauterine cannabis exposure leads to more aggressive behavior and attention problems in 18-month-old girls. *Drug Alcohol. Dep.* 118 470–474. 10.1016/j.drugalcdep.2011.03.004 21470799

[B33] EMCDDA (2019). *European Drug Report. Trends and Developments. European Monitoring Centre for Drugs and Drug Addiction* [preprint]. Available online at: http://www.emcdda.europa.eu/system/files/publications/11364/20191724_TDAT19001ENN_PDF.pdf (accessed December 12, 2019).

[B34] England L. J. Aagaard K. Bloch M. Conway K. Cosgrove K. Grana R. (2017). Developmental toxicity of nicotine: a transdisciplinary synthesis and implications for emerging tobacco products. *Neurosci. Biobehav. Rev.* 72 176–189. 10.1016/j.neubiorev.2016.11.013 27890689 PMC5965681

[B35] Eriksson M. Zetterström R. (1994). Amphetamine addiction during pregnancy: 10-year follow-up. *Acta Paediatr.* 83 27–31. 10.1111/j.1651-2227.1994.tb13380.x 7531039

[B36] Fergusson D. M. Woodward L. J. Horwood L. J. (1998). Maternal smoking during pregnancy and psychiatric adjustment in late adolescence. *Arch. Gen. Psychiatry* 55 721–727. 10.1001/archpsyc.55.8.721 9707383

[B37] Fernandez-Ruiz J. Bonnin A. De Miguel R. Castro J. G. Ramos J. A. (1998). Peinatal exposure to marihuana or its main psychotive constituent, delta9-tetrahydrocannabinol, affects the development of brain dopaminergic nerons. *Arq. Med.* 12 67–77.

[B38] Fine J. D. Moreau A. L. Karcher N. R. Agrawal A. Rogers C. E. Barch D. M. (2019). Association of prenatal cannabis exposure with psychosis proneness among children in the adolescent brain cognitive development (ABCD) study. *JAMA Psychiatry* 76 762–764. 10.1001/jamapsychiatry.2019.0076 30916716 PMC6583849

[B39] Forray A. (2016). Substance use during pregnancy. *F1000Res* 5:F1000 Faculty Rev-887.

[B40] Fox D. J. Pettygrove S. Cunniff C. O’Leary L. A. Gilboa S. M. Bertrand J. (2015). Fetal alcohol syndrome among children aged 7–9 years—Arizona. Colorado, and New York, 2010. *MMWR* 64 54–57. 25632951 PMC4584557

[B41] Fried P. A. Makin J. E. (1987). Neonatal behavioural correlates of prenatal exposure to marihuana, cigarettes and alcohol in a low risk population. *Neurotoxicol. Teratol.* 9 1–7. 10.1016/0892-0362(87)90062-6 3627073

[B42] Fried P. A. Smith A. M. (2001). A literature review of the consequences of prenatal marihuana exposure: an emerging theme of a deficiency in aspects of executive function. *Neurotoxicol. Teratol.* 23 1–11. 10.1016/s0892-0362(00)00119-7 11274871

[B43] Gatzke-Kopp L. M. Beauchaine T. P. (2007). Direct and passive prenatal nicotine exposure and the development of externalizing psychopathology. *Child. Psychiatry Hum. Dev.* 38 255–269. 10.1007/s10578-007-0059-4 17520361 PMC2711763

[B44] Gkioka E. Korou L. M. Daskalopoulou A. Misitzi A. Batsidis E. Bakoyiannis I. (2016). Prenatal cocaine exposure and its impact on cognitive functions of offspring: a pathophysiological insight. *Rev. Neurosci.* 27 523–534. 10.1515/revneuro-2015-0064 26953708

[B45] Gomez-Pomar E. Finnegan L. P. (2018). The epidemic of neonatal abstinence syndrome, historical references of Its’. Origins, assessment, and management. *Front. Pediatr.* 6:33. 10.3389/fped.2018.00033 29520355 PMC5827164

[B46] Greenfield S. F. Back S. E. Lawson K. Brady K. T. (2010). Substance abuse in women. *Psychiatr. Clin. North Am.* 33 339–355. 10.1016/j.psc.2010.01.004 20385341 PMC3124962

[B47] Haight S. C. Ko J. Y. Tong V. T. Bohm M. K. Callaghan W. M. (2018). Opioid use disorder documented at delivery hospitalization - United States, 1999-2014. *MMWR* 67 845–849. 10.15585/mmwr.mm6731a1 30091969 PMC6089335

[B48] Hales C. N. Barker D. J. (2001). The thrifty phenotype hypothesis. *Br. Med. Bull.* 60 5–20. 10.1093/bmb/60.1.5 11809615

[B49] Haley D. W. Handmaker N. S. Lowe J. (2006). Infant stress reactivity and prenatal alcohol exposure. *Alcohol. Clin. Exp. Res.* 30 2055–2064. 10.1111/j.1530-0277.2006.00251.x 17117971

[B50] Herman L. E. Acosta M. C. Chang P. N. (2008). Gender and attention deficits in children diagnosed with a fetal alcohol spectrum disorder. *Can. J. Clin. Pharmacol.* 15 411–419. 18953085

[B51] Howell K. K. Lynch M. E. Platzman K. A. Smith G. H. Coles C. D. (2006). Prenatal alcohol exposure and ability, academic achievement, and school functioning in adolescence: a longitudinal follow-up. *J. Pediatr. Psychol.* 31 116–126. 10.1093/jpepsy/jsj029 15829611

[B52] Hurd Y. L. Manzoni O. J. Pletnikov M. V. Lee F. S. Bhattacharyya S. Melis M. (2019). Cannabis and the developing brain: Insights into its long-lasting effects. *J. Neurosci.* 39 8250–8258. 10.1523/jneurosci.1165-19.2019 31619494 PMC6794936

[B53] Hutchinson J. Pickett K. E. Green J. Wakschlag L. S. (2010). Smoking in pregnancy and disruptive behaviour in 3-year-old boys and girls: an analysis of the UK Millennium Cohort Study. *J. Epidemiol. Community Health* 64 82–88. 10.1136/jech.2009.089334 19887578 PMC10088058

[B54] Janssen B. G. Byun H.-M. Gyselaers W. Lefebvre W. Baccarelli A. A. Nawrot T. S. (2015). Placental mitochondrial methylation and exposure to airborne particulate matter in the early life environment: an ENVIR ON AGE birth cohort study. *Epigenetics* 10 536–544. 10.1080/15592294.2015.1048412 25996590 PMC4623402

[B55] Jansson L. M. Dipietro J. A. Elko A. Velez M. (2007). Maternal vagal tone change in response to methadone is associated with neonatal abstinence syndrome severity in exposed neonates. *J. Matern. Fetal Neonatal Med.* 20 677–685. 10.1080/14767050701490327 17701668

[B56] Jansson L. M. Dipietro J. A. Elko A. Velez M. (2010). Infant autonomic functioning and neonatal abstinence syndrome. *Drug Alcohol Depend.* 109 198–204. 10.1016/j.drugalcdep.2010.01.004 20189732 PMC2875284

[B57] Jantzen K. Ball S. A. Leventhal J. M. Schottenfeld R. S. (1998). Types of abuse and cocaine use in pregnant women. *J. Subst. Abuse Treat.* 15 319–323. 10.1016/s0740-5472(97)00198-0 9650140

[B58] Karmel B. Z. Gardner J. M. (1996). Prenatal cocaine exposure effects on arousal-modulated attention during the neonatal period. *Dev. Psychobiol.* 29 463–480. 10.1002/(sici)1098-2302(199607)29:5<463::aid-dev5>3.0.co;2-m 8809496

[B59] Kelly Y. Sacker A. Gray R. Kelly J. Wolke D. Quigley M. A. (2009). Light drinking in pregnancy, a risk for behavioural problems and cognitive deficits at 3 years of age? *Int. J. Epidemiol.* 38 129–140. 10.1093/ije/dyn230 18974425

[B60] Keyes K. M. Hatzenbuehler M. L. McLaughlin K. A. Link B. Olfson M. Grant B. F. (2010). Stigma and treatment for alcohol disorders in the United States. *Am. J. Epidemiol.* 172 1364–1372. 10.1093/aje/kwq304 21044992 PMC2998202

[B61] Keyes K. M. Martins S. S. Hasin D. S. (2008). Past 12-month and lifetime comorbidity and poly-drug use of ecstasy users among young adults in the United States: results from the National Epidemiologic Survey on Alcohol and Related Conditions. *Drug Alcohol Depend.* 97 139–149. 10.1016/j.drugalcdep.2008.04.001 18524499 PMC3771490

[B62] Khalife N. Glover V. Hartikainen A.-L. Taanila A. Ebeling H. Järvelin M.-R. (2012). Placental size is associated with mental health in children and adolescents. *PLoS One* 7:e40534. 10.1371/journal.pone.0040534 22792364 PMC3392232

[B63] Khan A. Faucett J. Morrison S. Brown W. A. (2013). Comparative mortality risk in adult patients with schizophrenia, depression, bipolar disorder, anxiety disorders, and attention-deficit/hyperactivity disorder participating in psychopharmacology clinical trials. *JAMA Psychiatry* 70 1091–1099. 10.1001/jamapsychiatry.2013.149 23986353

[B64] Kiblawi Z. N. Smith L. M. Diaz S. D. LaGasse L. L. Derauf C. Newman E. (2014). Prenatal methamphetamine exposure and neonatal and infant neurobehavioral outcome: results from the IDEAL study. *Subst. Abus.* 35 68–73. 10.1080/08897077.2013.814614 24588296 PMC3942806

[B65] Kiblawi Z. N. Smith L. M. LaGasse L. L. Derauf C. Newman E. Shah R. (2013). The effect of prenatal methamphetamine exposure on attention as assessed by continuous performance tests: Results from the infant development, environment, and lifestyle (IDEAL) study. *J. Dev. Behav. Pediatr.* 34 31–37. 10.1097/DBP.0b013e318277a1c5 23275056 PMC3800474

[B66] Kodituwakku P. W. (2007). Defining the behavioral phenotype in children with fetal alcohol spectrum disorders: a review. *Neurosci. Biobehav. Rev.* 31 192–201. 10.1016/j.neubiorev.2006.06.020 16930704

[B67] Kotimaa A. J. Moilanen I. Taanila A. Ebeling H. Smalley S. L. McGough J. J. (2003). Maternal smoking and hyperactivity in 8-year-old children. *J. Am. Acad. Child Adolesc. Psychiatry* 42 826–833. 10.1097/01.chi.0000046866.56865.a2 12819442

[B68] Kratimenos P. Penn A. A. (2019). Placental programming of neuropsychiatric disease. *Pediatr. Res.* 86 157–164. 10.1038/s41390-019-0405-9 31003234 PMC11906117

[B69] LaGasse L. L. Derauf C. Smith L. M. Newman E. Shah R. Neal C. (2012). Prenatal methamphetamine exposure and childhood behavior problems at 3 and 5 years of age. *Pediatrics* 129 681–688. 10.1542/peds.2011-2209 22430455 PMC3313637

[B70] Langley K. Holmans P. A. van den Bree M. B. Thapar A. (2007). Effects of low birth weight, maternal smoking in pregnancy and social class on the phenotypic manifestation of Attention Deficit Hyperactivity Disorder and associated antisocial behaviour: investigation in a clinical sample. *BMC Psychiatry* 7:26. 10.1186/1471-244x-7-26 17584500 PMC1913513

[B71] Leech S. L. Larkby C. A. Day R. Day N. L. (2006). Predictors and correlates of high levels of depression and anxiety symptoms among children at age 10. *J. Am. Acad. Child. Adolesc. Psychiatry* 45 223–230. 10.1097/01.chi.0000184930.18552.4d 16429093

[B72] Lu L. H. Johnson A. O’Hare E. D. Bookheimer S. Y. Smith L. M. O’Connor M. J. (2009). Effects of prenatal methamphetamine exposure on verbal memory revealed with fMRI. *J. Dev. Behav. Pediatr.* 30 185–192. 10.1097/DBP.0b013e3181a7ee6b 19525715 PMC2745202

[B73] Mannon E. C. Ray S. C. Ryan M. J. Sullivan J. C. (2020). Does sex matter: an update on the implementation of sex as a biological variable in research. *Am. J. Physiol. Renal Physiol.* 318 F329–F331. 31904284 10.1152/ajprenal.00575.2019 PMC7052660

[B74] Mattson S. N. Riley E. P. Gramling L. Delis D. C. Jones K. L. (1998). Neuropsychological comparison of alcohol-exposed children with or without physical features of fetal alcohol syndrome. *Neuropsychology* 12 146–153. 10.1037/0894-4105.12.1.146 9460742

[B75] May P. A. Baete A. Russo J. Elliott A. J. Blankenship J. Kalberg W. O. (2014). Prevalence and characteristics of fetal alcohol spectrum disorders. *Pediatrics* 134 855–866. 10.1542/peds.2013-3319 25349310 PMC4210790

[B76] May P. A. Gossage J. P. Marais A. S. Adnams C. M. Hoyme H. E. Jones K. L. (2007). The epidemiology of fetal alcohol syndrome and partial FAS in a South African community. *Drug Alcohol. Depend.* 88 259–271. 10.1016/j.drugalcdep.2006.11.007 17127017 PMC1865526

[B77] Mayes L. C. (1996). Exposure to cocaine: behavioral outcomes in preschool and school-age children. *NIDA Res. Monogr.* 164 211–229. 8809873

[B78] Mayes L. C. Cicchetti D. Acharyya S. Zhang H. (2003). Developmental trajectories of cocaine-and-other-drug-exposed and non-cocaine-exposed children. *J. Dev. Behav. Pediatr.* 24 323–335. 10.1097/00004703-200310000-00003 14578693

[B79] Mayes L. C. Grillon C. Granger R. Schottenfeld R. (1998). Regulation of arousal and attention in preschool children exposed to cocaine prenatally. *Ann. N. Y. Acad. Sci.* 846 126–143. 10.1111/j.1749-6632.1998.tb09731.x 29087549

[B80] McHugh R. K. DeVito E. E. Dodd D. Carroll K. M. Potter J. S. Greenfield S. F. (2013). Gender differences in a clinical trial for prescription opioid dependence. *J. Subst. Abuse Treat.* 45 38–43. 10.1016/j.jsat.2012.12.007 23313145 PMC3626739

[B81] Moe V. Slinning K. (2001). Children prenatally exposed to substances: Gender-related differences in outcome from infancy to 3 years of age. *Infant Mental Health J.* 22 334–350. 10.1002/imhj.1005

[B82] Noland J. S. Singer L. T. Short E. J. Minnes S. Arendt R. E. Kirchner H. L. (2005). Prenatal drug exposure and selective attention in preschoolers. *Neurotoxicol. Teratol.* 27 429–438. 10.1016/j.ntt.2005.02.001 15939203

[B83] Nordstrom Bailey B. Sood B. G. Sokol R. J. Ager J. Janisse J. Hannigan J. H. (2005). Gender and alcohol moderate prenatal cocaine effects on teacher-report of child behavior. *Neurotoxicol. Teratol.* 27 181–189. 10.1016/j.ntt.2004.10.004 15734269

[B84] Nygaard E. Moe V. Slinning K. Walhovd K. B. (2015). Longitudinal cognitive development of children born to mothers with opioid and polysubstance use. *Pediatr. Res.* 78 330–335. 10.1038/pr.2015.95 25978800 PMC4539602

[B85] Nygaard E. Slinning K. Moe V. Walhovd K. B. (2017). Cognitive function of youths born to mothers with opioid and poly-substance abuse problems during pregnancy. *Child Neuropsychol.* 23 159–187. 10.1080/09297049.2015.1092509 26471942

[B86] Ornoy A. Ergaz Z. (2010). Alcohol abuse in pregnant women: effects on the fetus and newborn, mode of action and maternal treatment. *Int. J. Environ. Res. Public Health* 7 364–379. 10.3390/ijerph7020364 20616979 PMC2872283

[B87] Ornoy A. Segal J. Bar-Hamburger R. Greenbaum C. (2001). Developmental outcome of school-age children born to mothers with heroin dependency: importance of environmental factors. *Dev. Med. Child. Neurol.* 43 668–675. 10.1017/s0012162201001219 11665823

[B88] Park B. Y. Misra D. P. Moye J. Miller R. K. Croen L. Fallin M. D. (2018). Placental gross shape differences in a high autism risk cohort and the general population. *PLoS One* 13:e0191276. 10.1371/journal.pone.0191276 30133439 PMC6104917

[B89] Parker S. E. Mai C. T. Canfield M. A. Rickard R. Wang Y. Meyer R. E. (2010). Updated National Birth Prevalence estimates for selected birth defects in the United States, 2004-2006. *Birth Defects Res. A Clin. Mol. Teratol.* 88 1008–1016. 10.1002/bdra.20735 20878909

[B90] Patrick S. W. Cooper W. O. Davis M. M. (2017). Prescribing opioids and psychotropic drugs in pregnancy. *BMJ* 358:3616. 10.1136/bmj.j3616 PMC5538710 28768614

[B91] Paul S. E. Hatoum A. S. Fine J. D. Johnson E. C. Hansen I. Karcher N. R. (2019). Prenatal cannabis exposure and childhood outcomes: Results from the ABCD study. *medRxiv* [Preprint]. 10.1101/2019.12.18.19015164 PMC7512132 32965490

[B92] Pickett K. E. Wood C. Adamson J. D’Souza L. Wakschlag L. S. (2008). Meaningful differences in maternal smoking behaviour during pregnancy: implications for infant behavioural vulnerability. *J. Epidemiol. Community Health* 62 318–324. 10.1136/jech.2006.058768 18339824 PMC10087306

[B93] Piper B. J. Acevedo S. F. Kolchugina G. K. Butler R. W. Corbett S. M. Honeycutt E. B. (2011). Abnormalities in parentally rated executive function in methamphetamine/polysubstance exposed children. *Pharmacol. Biochem. Behav.* 98 432–439. 10.1016/j.pbb.2011.02.013 21334365 PMC3069661

[B94] Richardson G. A. Day N. L. Taylor P. M. (1989). The effect of prenatal alcohol, marijuana, and tobacco exposure on neonatal behavior. *Infant Behav. Dev.* 12 199–209. 10.1016/0163-6383(89)90006-4

[B95] Richardson G. A. Ryan C. Willford J. Day N. L. Goldschmidt L. (2002). Prenatal alcohol and marijuana exposure: effects on neuropsychological outcomes at 10 years. *Neurotoxicol. Teratol.* 24 309–320. 10.1016/s0892-0362(02)00193-9 12009486

[B96] Riley E. P. McGee C. L. (2005). Fetal alcohol spectrum disorders: an overview with emphasis on changes in brain and behavior. *Exp. Biol. Med.* 230 357–365. 10.1177/15353702-0323006-03 15956765

[B97] Roescher A. M. Timmer A. Erwich J. J. H. Bos A. F. (2014). Placental pathology, perinatal death, neonatal outcome, and neurological development: a systematic review. *PLoS One* 9:e89419. 10.1371/journal.pone.0089419 24586764 PMC3934891

[B98] Ross E. J. Graham D. L. Money K. M. Stanwood G. D. (2015). Developmental consequences of fetal exposure to drugs: what we know and what we still must learn. *Neuropsychopharmacology* 40 61–87. 10.1038/npp.2014.147 24938210 PMC4262892

[B99] Rydell M. Cnattingius S. Granath F. Magnusson C. Galanti M. R. (2012). Prenatal exposure to tobacco and future nicotine dependence: population-based cohort study. *Br. J. Psychiatry* 200 202–209. 10.1192/bjp.bp.111.100123 22322457

[B100] SAMHSA (2011). *Results from the 2010 National Survey on Drug Use and Health: Summary of National Findings. U.S. DEPARTMENT OF HEALTH AND HUMAN SERVICES, Substance Abuse and Mental Health Services Administration, Center for Behavioral Health Statistics and Quality [Preprint].* Available online at: http://www.samhsa.gov/data/sites/default/files/cbhsq-reports/NSDUHFFR2017/NSDUHFFR2017.pdf (accessed March 8, 2020).

[B101] SAMHSA (2018). *Key Substance Use and Mental Health Indicators in the United States: Results from the 2017 National Survey on Drug Use and Health. U.S. DEPARTMENT OF HEALTH AND HUMAN SERVICES, Substance Abuse and Mental Health Services Administration, Center for Behavioral Health Statistics and Quality [Preprint].* Available online at: http://www.samhsa.gov/data/sites/default/files/cbhsq-reports/NSDUHFFR2017/NSDUHFFR2017.pdf (accessed March 8, 2020).

[B102] Scheyer A. F. Melis M. Trezza V. Manzoni O. J. (2019). Consequences of perinatal cannabis exposure. *Trends Neurosci.* 42 871–884. 10.1016/j.tins.2019.08.010 31604585 PMC6981292

[B103] Schneider M. L. Moore C. F. Adkins M. M. (2011). The effects of prenatal alcohol exposure on behavior: rodent and primate studies. *Neuropsychol. Rev.* 21 186–203. 10.1007/s11065-011-9168-8 21499982 PMC4226068

[B104] Seedat S. Scott K. M. Angermeyer M. C. Berglund P. Bromet E. J. Brugha T. S. (2009). Cross-national associations between gender and mental disorders in the World Health Organization World Mental Health Surveys. *Arch. Gen. Psychiatry* 66 785–795. 10.1001/archgenpsychiatry.2009.36 19581570 PMC2810067

[B105] Shah R. Diaz S. D. Arria A. LaGasse L. L. Derauf C. Newman E. (2012). Prenatal methamphetamine exposure and short-term maternal and infant medical outcomes. *Am. J. Perinatol.* 29 391–400. 10.1055/s-0032-1304818 22399214 PMC3717348

[B106] Singer L. T. Minnes S. Short E. Arendt R. Farkas K. Lewis B. (2004). Cognitive outcomes of preschool children with prenatal cocaine exposure. *JAMA* 291 2448–2456. 15161895 10.1001/jama.291.20.2448 PMC10249064

[B107] Smith A. M. Fried P. A. Hogan M. J. Cameron I. (2004). Effects of prenatal marijuana on response inhibition: an fMRI study of young adults. *Neurotoxicol. Teratol.* 26 533–542. 10.1016/j.ntt.2004.04.004 15203175

[B108] Smith A. M. Fried P. A. Hogan M. J. Cameron I. (2006). Effects of prenatal marijuana on visuospatial working memory: an fMRI study in young adults. *Neurotoxicol. Teratol.* 28 286–295. 10.1016/j.ntt.2005.12.008 16473495

[B109] Sood B. G. Nordstrom Bailey B. Covington C. Sokol R. J. Ager J. Janisse J. (2005). Gender and alcohol moderate caregiver reported child behavior after prenatal cocaine. *Neurotoxicol. Teratol.* 27 191–201. 10.1016/j.ntt.2004.10.005 15734270

[B110] Sourander A. Sucksdorff M. Chudal R. Surcel H.-M. Hinkka-Yli-Salomäki S. Gyllenberg D. (2019). Prenatal cotinine levels and ADHD among offspring. *Pediatrics* 143:e20183144. 10.1542/peds.2018-3144 30804074 PMC6398365

[B111] Steingrimsson S. Carlsen H. K. Sigfusson S. Magnusson A. (2012). The changing gender gap in substance use disorder: a total population-based study of psychiatric in-patients. *Addiction* 107 1957–1962. 10.1111/j.1360-0443.2012.03954.x 22632169

[B112] Suffet F. Brotman R. (1984). A comprehensive care program for pregnant addicts: obstetrical, neonatal, and child development outcomes. *Int. J. Addict.* 19 199–219. 10.3109/10826088409057176 6724763

[B113] Thanh N. X. Jonsson E. Salmon A. Sebastianski M. (2014). Incidence and prevalence of fetal alcohol spectrum disorder by sex and age group in Alberta. Canada. *J. Popul. Ther. Clin. Pharmacol.* 21 395–404. 25381628

[B114] Treit S. Lebel C. Baugh L. Rasmussen C. Andrew G. Beaulieu C. (2013). Longitudinal MRI reveals altered trajectory of brain development during childhood and adolescence in fetal alcohol spectrum disorders. *J. Neurosci.* 33 10098–10109. 10.1523/JNEUROSCI.5004-12.2013 23761905 PMC6618394

[B115] Wakschlag L. S. Hans S. L. (2002). Maternal smoking during pregnancy and conduct problems in high-risk youth: a developmental framework. *Dev. Psychopathol.* 14 351–369. 10.1017/s0954579402002092 12030696

[B116] Wakschlag L. S. Lahey B. B. Loeber R. Green S. M. Gordon R. A. Leventhal B. L. (1997). Maternal smoking during pregnancy and the risk of conduct disorder in boys. *Arch. Gen. Psychiatry* 54 670–676. 10.1001/archpsyc.1997.01830190098010 9236551

[B117] Walsh K. McCormack C. A. Webster R. Pinto A. Lee S. Feng T. (2019). Maternal prenatal stress phenotypes associate with fetal neurodevelopment and birth outcomes. *Proc. Natl. Acad. Sci. U.S.A.* 116 23996–24005. 10.1073/pnas.1905890116 31611411 PMC6883837

[B118] Wilcox J. A. Yates W. R. (1993). Gender and psychiatric comorbidity in substance-abusing individuals. *Am. J. Addict.* 2 202–206. 10.3109/10550499309113939

[B119] Willford J. A. Richardson G. A. Leech S. L. Day N. L. (2004). Verbal and visuospatial learning and memory function in children with moderate prenatal alcohol exposure. *Alcohol. Clin. Exp. Res.* 28 497–507. 10.1097/01.alc.0000117868.97486.2d 15084908

[B120] Willoughby M. Greenberg M. Blair C. Stifter C. (2007). Neurobehavioral consequences of prenatal exposure to smoking at 6 to 8 months of age. *Infancy* 12 273–301. 10.1111/j.1532-7078.2007.tb00244.x

[B121] Zabaneh R. Smith L. M. LaGasse L. L. Derauf C. Newman E. Shah R. (2012). The effects of prenatal methamphetamine exposure on childhood growth patterns from birth to 3 years of age. *Am. J. of Perinatal.* 29 203–210. 10.1055/s-0031-1285094 21818727 PMC3717349

